# Off-label drug use in China after the Physician Law (2021): legal challenges and solutions

**DOI:** 10.3389/fphar.2025.1547418

**Published:** 2025-04-14

**Authors:** Shenbao Liang, Fanxian Cai

**Affiliations:** School of Law, Shanghai University of Finance and Economics, Shanghai, China

**Keywords:** off-label drug use, evidence-based support, informed consent, internal review procedure, medical liability

## Abstract

**Introduction:**

In 2021 China enacted the Physician Law, which specifically outlines the legal requirements for off-label drug use in Article 29. Legislators, medical institutions, and judicial authorities all hoped that this new regulation would effectively reduce illegal off-label drug use. However, many violations still occurred in medical practice even after the enactment of the Physician Law, leading to judicial cases. This article examines the current state of off-label drug use in China, and the legal liability risks faced by medical institutions.

**Methods:**

This study investigates data from two main aspects: (1) judicial decisions related to off-label drug use; and (2) surveys associated with off-label drug use from medical institutions. Systematic literature search and descriptive data analysis was conducted in this paper.

**Results:**

This article identifies three main forms of non-compliant off-label drug use: first, insufficient evidence-based justification; second, deficiencies in the informed consent process; and third, the lack of an internal review system. These are also the primary factors leading to legal liability for medical institutions.

**Discussion:**

From a legal doctrinal perspective, the article concludes by elaborating on how each legal requirement should be met. Our research provides insights into the factors that lead to legal liability for off-label drug use and examines how physicians and medical institutions can avoid such liability.

## 1 Introduction

Off-label drug use—the prescription of medications in ways not specified in their official labeling—is a pervasive phenomenon in global healthcare. In the United States, for example, it is estimated that off-label prescriptions constitute approximately 21% of all medication use (Radley et al., 2006). In the European Union, meanwhile, off-label drug use is widespread across member states, although the proportion may vary between countries. For instance, in France, the number of neonatal prescriptions that are off-label may range from 55% to 80% (Riou et al., 2015). This significant reliance on off-label prescribing highlights the universal challenges faced by physicians when approved treatments do not fully address patient needs.

There are different types of off-label drug uses. The most common categories include off-label uses by indication, age, dosage, route of administration, formulation, and patient population (Mengato et al., 2025). The proportion of occurrence varies across different types of off-label uses. For instance, a study categorizing off-label drug use in a particular hospital over a certain period found that 67.9% of the off-label uses were due to indication, 49.6% were related to dosage deviations, and 44.4% were associated with age discrepancies. Furthermore, 52.4% of off-label uses were attributed to multiple factors (Mengato et al., 2025). Additionally, studies have shown that off-label drug use is relatively more prevalent in the fields of pediatrics and oncology. In pediatric medicine, where ethical barriers limit clinical trials, off-label prescriptions can account for as many as 70% of treatments (Kalko et al., 2021). This highlights a critical disparity between the available approved medications and the actual therapeutic needs of children. In the field of oncology medical treatments evolve rapidly, often outpacing regulatory approvals. As a result, off-label drug use can become necessary as physicians seek to provide patients with the latest, potentially life-saving therapies (Meng et al., 2022).

Internationally, the legal regulations surrounding off-label drug use differ widely. Countries such as the United States, Japan, France, and the United Kingdom have developed specific regulations that allow off-label prescribing under particular conditions, aiming to strike a balance between fostering medical innovation and ensuring patient safety (Weda et al., 2017). In Italy, both national legislation and AIFA guidelines govern off-label drug use. These regulations emphasize patient safety, informed consent, the need for a strong evidence base, and procedural transparency (Addis A., 2021). Some nations impose strict documentation and evidence requirements, while others grant physicians more flexibility and place greater trust in clinical judgment.

In contrast, China’s approach to off-label drug use has been less defined, resulting in significant legal ambiguity. Studies have reported that China’s off-label prescribing rates reached approximately 30.0% for anti-tumor drugs (Meng et al., 2022), 53.0%–82.7% for pediatric outpatients, 46.9%–95.0% for pediatric inpatients (Mei et al., 2017), and 7.5%–40.0% in general adult medications (Ding and Shao, 2022). Although off-label drug use was clearly widespread, the lack of clear legal guidelines created an uncertain environment for healthcare providers, wherein physicians, driven by their ethical duty to ease patient suffering and offer the best possible care, often resorted to off-label prescriptions. However, without statutory protection, they faced significant legal risks. This uncertainty led to a rise in medical liability cases, strained relationships between doctors and patients, and eroded trust in the healthcare system (Si and Ma, 2023).

Recognizing these challenges, the People’s Republic of China took a significant legislative step by enacting the Physician Law on 20 August 2021. Article 29, Paragraph 2, of this law explicitly permits off-label drug use under defined conditions, establishing four core preconditions for lawful practice: (1) necessity, indicating no effective or better treatment alternatives are available; (2) suitability, requiring sufficient support from evidence-based medicine; (3) informed consent from the patient; and (4) internal review and approval by the medical institution.

However, it is now critical to assess compliance with off-label drug use regulations in China. Although the law provides a clearer framework, some legal requirements continue to be violated in medical practice, exposing hospitals to liability in doctor-patient lawsuits.

By reviewing judicial decisions and analyzing medical survey data, this paper aims to illustrate the current status of off-label drug use in China and identify key factors leading to medical liability, from both the medical and judicial perspectives. Based on these findings, the paper interprets the legal preconditions for off-label drug use, with the goal of enhancing healthcare professionals’ awareness of compliance, thereby reducing legal risks.

## 2 Method

This study investigates data from two main aspects: (1) judicial decisions related to off-label drug use; and (2) surveys associated with off-label drug use from medical institutions. We searched for judicial decisions in the legal database without restricting the time period, meaning that any judicial case matching the search keywords was included. For survey studies on off-label drug use, we limited the search to publications after 2021. The search and analysis were conducted between 8 November 2024, and 18 December 2024.

### 2.1 Judicial decisions collected

We searched for judicial decisions across multiple databases, including PKULAW, Faxin, and Wenshu. court.gov.cn, which are the three main platforms for Chinese judicial decisions. After searching and reviewing the data, we found that PKULAW contained the most comprehensive set of cases. The search results from PKULAW also encompassed those from the other two platforms. Therefore, we decided to use PKULAW as the sole source for judicial decisions.

We searched the legal database “PKULAW” using “off-label drug use” and its synonyms as keywords, resulting in 58 court judgments. Upon reviewing the content of these judgments, we found that 12 cases were unrelated to our research topic; these cases primarily concerned whether the costs of off-label drug use could be reimbursed by insurance. The remaining 46 cases were relevant to our study. We conducted a statistical analysis of the reasoning in these 46 judgments to identify the main causes of liability associated with off-label drug use. Furthermore, we compare the number of cases before and after the enactment of the Physician Law.

### 2.2 Survey data from medical institutions collected

To assess the current state of off-label drug use in medical practice, we summarized findings from publicly published survey studies.

#### 2.2.1 Search strategy

We conducted a systematic literature search *via* CNKI (China National Knowledge Infrastructure), SinoMed, PubMed, EMbase and WOS (web of science). These five databases are most frequently used to search papers among medical researchers. The search terms included “off-label drug use”, “unlicensed use” and other similar terms. The date of publication included is between 20 August 2021 (when the Phsician law enacted) and 18 December 2024 or from 2021 to 2024.

#### 2.2.2 Inclusion and exclusion criteria

The study included documents relevant to off-label drug use in China. They could comprehensively cover the topic of off-label drug use or focus on the specific aspects, such as prevalence of off-label drug use, evidence evaluation, informed consent or internal review procedure. The exclusion criteria were as follows: 1. not about Chinese but other countries’ situation; 2. pure review studies or pure theoretical discussion that lacks survey data; 3. published after 20 August 2021, but the data sources are from before 20 August 2021. Excluded also the documents with data sources spanning the time before and after the enactment of the Physician Law, but with a reservation of comparing data before and after the enactment of the Physician Law; 4. In cases where multiple documents based on the same survey data, only the earliest published paper was included.

The literature search was independently conducted by two authors to avoid the influence of subjective bias on the search results. Any discrepancies were resolved through discussion. A total of 28 qualified articles were obtained.

**FIGURE 1:**
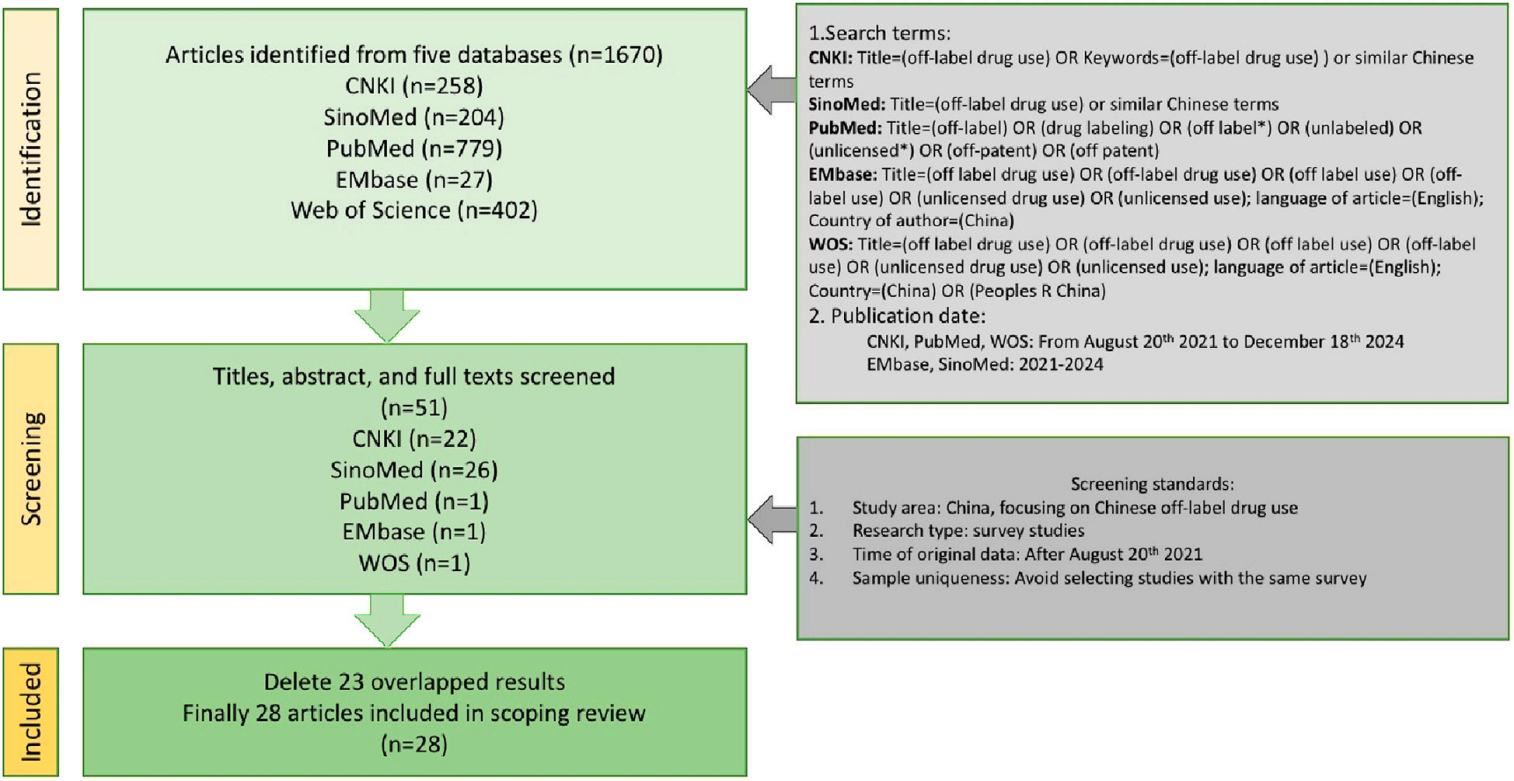
Flow diagram of the literature search.

The literature search process is illustrated in Figure 1.

#### 2.2.3 Data extraction

The two authors read these 28 articles and identified the following four aspects of information: 1. prevalence of off-label drug use; 2. Evaluation of evidence-based support; 3. Situation of informed consent; 4. Situation of internal review procedure. Any discrepancies were resolved through discussion. These studies provided insights into the prevalence and factors of non-compliance in off-label drug use within medical institutions. But not every article covers all of the above four points.

#### 2.2.4 Data analysis

Descriptive data analysis was conducted in this paper, focusing solely on presenting the data in a clear and concise manner, without testing hypotheses or making predictions. The objective of the literature analysis in this manuscript is to provide an overview of current medical practices. We present the proportions of off-label drug use, the availability of sufficient evidence-based support, the implementation of informed consent, and the establishment of internal review procedures. Based on these findings, we conclude the legal challenges and discuss potential solutions.

## 3 Result

### 3.1 Analysis of judicial decision data

Among the 46 cases analyzed from 2014 to 2024, five resulted in no liability for damages, while 41 held medical institutions liable. Of these, 40 cases occurred and were adjudicated before the enactment of the Physician Law, 1 case occurred before but was decided after its enactment, and 5 cases both occurred and were adjudicated after the enactment of the Physician law (Table 1).

**TABLE 1:** Liability outcomes before and after the enactment of the physician law (2014–2024).

Time period	Number of cases	Medical institutions held liable	Medical institutions not liable
Occurred and adjudicated before the Physician Law	40	35	5
Occurred before the Physician Law, but adjudicated after	1	1	0
Occurred and adjudicated after the Physician Law	5	5	0
Total	46	41	5

The types of non-compliance related to off-label drug use in these 41 cases were categorized as follows (see Table 2): lack of evidence-based support; violation of informed consent rules; and deficiencies in internal review procedures. The judges did not consider the precondition in Article 29, Paragraph two of the Physician Law which states that off-label drug use is permissible only when “no effective or better treatment methods are available.”

**TABLE 2:** Types of non-compliance leading to liability (2014–2024**)**.

Type of non-compliance	Number of cases
Lack of evidence-based support	15
Violation of informed consent rules	11
Deficiencies in internal review procedure	3
Unspecified violations	12
Total	41

In some cases the court judgments only mentioned medical malpractice and off-label drug use, without specifying which legal requirement was violated.

### 3.2 Results of medical practice

This section presents findings related to off-label drug use from several perspectives: the frequency of occurrence, the presence of evidence-based support, implementation of informed consent, and the establishment and execution of internal review procedures. Among the collected 28 articles, 26 address the issue of the frequency of occurrence, 11 address the issue of evaluation of evidence-based support, four address the issue of implementation of informed consent, and four address the issue of internal review procedure. We have presented the data corresponding to the first two issues in a single table, namely, Table 3, and the data corresponding to the last two issues in another table, namely, Table 4.

**TABLE 3:** Frequency of off-label drug use and Presence of evidence-based support (2021–2024).

No.	Resources	Prevalence of off-label drug use (%)			Evaluation of evidence from EBM (%)
1	Lin et al. (2024)	**Prevalence in medical institutions**		100.00	\
2	Song et al. (2024)			85.71	\
3	Zhang R. et al. (2024a)			49.16	\
4	Zhang R. et al. (2024b)			91.67	\
5	Zhang Y.D. et al. (2024)	**Proportion by medical orders**		2.25	\
6	Zhang Y.T. et al. (2024)			46.43	High level: 49.85 Middle level: 38.30 Low level: 11.85
7	Jia et al. (2023)			85.55	\
8	Shen et al. (2024)			83.59	For drugs that not on list: 40.62 insufficient
9	Quan et al. (2022)			12.89	\
10	Tan et al. (2023)	**Proportion by prescriptions**		2.57	19.27 insufficient
11	Lv et al. (2024)			38.69	22.12 insufficient
12	Gao M.M. et al. (2023)			6.30	\
13	Zhong et al. (2023)			62.36	\
14	Sun (2023)			13.39	\
15	Wang, X. (2024)			17.24	\
16	Yan et al. (2024)			16.88	\
17	Zhang G. J. et al. (2024)			5.06	62.09 insufficient
18	Luo (2024)	**Proportion by medical orders or by prescriptions**		off-label medical orders: 24.23 off-label prescriptions: 20.44	\
19	Huang et al. (2024)			off-label medical orders: 43.98 off-label prescriptions: 36.70	\
20	Li and Huang (2024a)	**Regarding specific drugs**	**by prescriptions**	off-label uses by indication: 25.09	Strong evidence: 38.40 Weak evidence:48.00
21	Li and Huang (2024b)			off-label uses by indication:8.03	Strong evidence: 24.72, weak evidence: 51.69
22	Chen et al. (2024)			off-label uses by indication:3.43	Strong evidence: 38.27 Weak evidence: 49.38
23	Yue et al. (2024)			96.00	100.00 insufficient
24	Lao et al. (2024)			47.44	\
25	Du et al. (2024)		**by medical orders**	100.00	1.30 insufficient
26	Hong et al. (2023)			83.00	\
27	Gao X. et al. (2024)	\			Outpatient: 2.51 insufficient Inpatiens: 9.03 insufficient
28	Meng M. et al. (2024)	\			\

The backslash (\) in this table indicates that the article does not mention the relevant information.

**TABLE 4:** Implementation of Informed consent and Internal review Procedure (2021–2024).

No.	Resources	Implemetation of informed consent (%)	Internal review procedure (%)
1	Lin et al. (2024)	68.00 fully implementated 32.00 not fully implementated	\
2	Song et al. (2024)	\	83.33
3	Zhang R. et al. (2024a)	63.37	48.43
4	Zhang R. et al. (2024b)	75.32	84.42
5	Meng M. et al. (2024)	65.00	49.40

#### 3.2.1 The frequency of off-label drug use after the enactment of the physician law

1. Prevalence in medical Institutions (see Table 3): four studies have reported the prevalence of off-label drug use in medical institutions. They calculate the proportion of hospitals with off-label drug use by dividing the number of hospitals reporting off-label drug use by the total number of hospitals surveyed. According to a 2024 survey (Lin et al., 2024), all 146 surveyed hospitals reported instances of off-label drug use to varying degrees. Another study (Song et al., 2024) found that 85.71% of the surveyed hospitals had off-label drug use. In a study (Zhang R. et al., 2024b) conducted in Guizhou Province, 77 out of 84 tertiary medical institutions (91.67%) engaged in off-label drug use. Similarly, when the survey scope was expanded to include secondary hospitals in Guizhou, 204 out of 415 medical institutions (49.16%) reported off-label drug use (Zhang Y. D. et al., 2024). Although there are significant differences in the data from these four surveys, they all indicate that off-label drug use is widespread in medical institutions.
2. Proportion of OLDU by medical orders or prescriptions (see Table 3): 22 studies investigated medical orders or prescriptions to determine the proportion of off-label drug use. These studies can be divided into two categories: one focuses on the general proportion of off-label drug use, without targeting a specific drug, referred to as general studies; the other focuses on off-label drug use for specific drugs. Among the 15 general studies, the proportion of off-label drug use varied significantly across different departments and medical institutions, ranging from 2.25% to 85.55% (Zhang Y.D. et al., 2024; Zhang Y.T. et al., 2024; Jia et al., 2023; Shen et al., 2024; Quan et al., 2022; Tan et al., 2023; Lv et al., 2024; Gao M.M. et al., 2023; Zhong et al., 2023; Luo, 2024; Huang et al., 2024).

Regarding off-label drug use for specific drugs, seven studies were conducted. Some studies calculated the proportion of off-label drug use for drugs beyond their approved indications, while others included all types of off-label drug use. The proportions varied widely depending on the drug. For example, the proportion of off-label use for dupilumab beyond its approved indications was 8.03% (Li and Huang, 2024b), while for IVIG (Intravenous Immunoglobulin), the proportion was 100.00% (Du et al., 2024). Between these extremes, the proportion for anlotinib was 25.09% (Li and Huang, 2024a), for doxycycline in pediatric outpatient departments, it was 47.44% (Lao et al., 2024), for montelukast sodium, it was 83.00% (Hong and Lin, 2023), for lenvatinib, it was 3.43% (Chen et al., 2024), and for Tripterygium hypoglaucum hutch tablets, it was 96.00% (Yue et al., 2024).

#### 3.2.2 The presence of evidence-based support

11 studies investigated evidence-based support for off-label drug use (See Table 3). Gao et al. (2024) reviewed off-label prescriptions in the Obstetrics Department of Lianyungang Maternal and Child Health Hospital from January to June in both 2021 and 2022. The review included 1,074 outpatient and 2,993 inpatient prescriptions in 2021 (before the enactment of the Physician Law), as well as 1,235 outpatient and 2,016 inpatient prescriptions in 2022 (after the enactment). The results showed that in 2021, the proportion of off-label drug use without sufficient evidence was 8.29% for outpatients and 30.00% for inpatients. By 2022, this proportion had decreased to 2.51% for outpatients and 9.03% for inpatients. The decrease in these percentages indicates improved compliance with evidence-based practices.

Meanwhile, during the period from July 2022 to March 2023, 11.85% of the off-label antineoplastic drug use in the Gynecology Department of Shanghai 10th People’s Hospital was classified as low-evidence off-label use (Zhang Y.T. et al., 2024).

Tan et al. (2024) found through their investigation that 19.27% of off-label drug use in a dermatology outpatient department lacked sufficient evidence. Lv et al. (2024) reported that 22.12% of antidepressant combinations were considered inappropriate in children and adolescents with mental disorders. Shen et al. (2024) found that 40.62% of off-label drug use without prior approval had insufficient evidence.

Zhang G. J. et al. (2024) found that 62.09% of off-label drug use in obstetrics and gynecology Department of Bengbu third People’s Hospital lacked sufficient evidence.

Studies on off-label drug use for specific drugs revealed varying levels of evidence-based support. For instance, among hospitalized patients, 48.00% of off-label use of anlotinib was supported by only weak evidence (Li and Huang, 2024a), 49.38% of off-label use of lenvatinib had weak evidence (Chen et al., 2024), and 51.69% of off-label use of dupilumab had weak evidence (Li and Huang, 2024b). The probability of insufficient evidence for the use of IVIG (Intravenous Immunoglobulin) was 1.30% (Du et al., 2024). For Tripterygium hypoglaucum Hutch tablets, 100.00% of the use lacked evidence-based support (Du et al., 2024).

#### 3.2.3 Implementation of informed consent

Four studies involve the informed consent rules for off-label drug use (see Table 4). According to Zhang R. et al. (2024a), among 415 surveyed hospitals, 263 required patients’ informed consent for off-label drug use (63.37%), while 152 did not have specific informed consent procedures (36.63%). When the survey (Zhang R. et al., 2024b) was limited to tertiary hospitals, the proportion of hospitals implementing informed consent increased to 75.32%, while the proportion not implementing it decreased to 24.68%. Meng M. et al. (2024) found that 65.00% of hospitals implemented informed consent procedures, while 35.00% did not have an informed consent system for off-label drug use.

Lin et al. (2024) found that even in hospitals with off-label drug use management mechanisms, deficiencies in informed consent practices still existed. Specifically, 68.00% of the hospitals required informed consent forms for all off-label uses, but 32.00% had not fully implemented informed consent procedures.

#### 3.2.4 Establishment of internal review procedure

Four studies have reported the proportion of hospitals that have established internal review procedures for off-label drug use (see Table 4). According to data from Zhang R. et al. (2024a), among the 415 hospitals surveyed, 48.43% had established an internal review procedures for off-label drug use, while 51.57% had not. The results from the other three studies showed that the proportions of hospitals with internal review procedures were 49.40% (Meng M. et al., 2024), 83.33% (Song et al., 2024), and 84.43% (Zhang R. et al., 2024b), respectively.

#### 3.2.5 Consideration of “no effective or better treatment methods”

None of the survey data addressed the precondition of “no effective or better treatment methods available”. This requirement is not considered in medical practice, aligning with findings from judicial practice.

## 4 Discussion

Despite the implementation of the Physician Law in August 2021, hospitals continue to face numerous compliance issues related to off-label drug use, leading to frequent liability in doctor-patient disputes. Judicial judgments reveal that non-compliance with the requirements specified in Article 29, Paragraph two of the **Physician Law**—particularly in the areas of **evidence-based support**, **informed consent**, and **internal review procedure**—often results in medical liability. Our findings indicate significant deficiencies in these three aspects within medical practice.

This article aims to provide physicians with a clearer framework for lawful off-label prescribing, in order to help them avoid legal liability. The following discussion focuses on three key preconditions of off-label drug use: **evidence-based support**, **informed consent**, and **internal review procedure**.

### 4.1 What kind of evidence-based support is required?

Article 29, Paragraph 2, of the Physicians Law mandates that off-label drug use must be grounded in evidence from evidence-based medicine. Off-label drug use is appropriate only when this requirement is met; otherwise, the drug use is improper, and the physician may be held liable for negligence. The term “evidence-based medicine” and information derived from such a practice are therefore essential criteria to judge whether a particular instance of off-label drug use is suitable or not. In the following we will explain what these factors are in the context of both medical and judicial practice.

#### 4.1.1 Evidence-based medicine

The concept of evidence-based medicine emerged in the 1980s. The clinical epidemiologist Professor David Sackett et al., 1996 defined it as “the conscientious, explicit, and judicious use of current best evidence in making decisions about the care of individual patients.” The core idea is that medical decisions should be grounded as much as possible in objective research findings. Evidence-based medicine transformed the traditional approach to treatment, which relied on physicians’ personal experiences, the guidance of senior physicians, and fragmented information from textbooks and select journals, into a model that depends on extensive publicly available clinical research data for diagnostic and therapeutic decisions (Tonelli and Shapiro, 2020).

The concept of evidence-based medicine has gained acceptance within the Chinese medical community (Wang and Jin, 2019). On one hand, medical schools require students specializing in clinical medicine to study evidence-based medicine as a mandatory part of their course. Throughout their studies students learn the concepts and best methods for seeking medical evidence. In clinical practice, on the other hand, evidence from evidence-based medicine is increasingly valued by clinicians, who use high-quality medical evidence to guide their clinical decisions.

These definitions raise some obvious questions: what types of evidence are used in evidence-based medicine, and how are they prioritized and graded?

#### 4.1.2 Evidence-based support from the medical perspective

When viewed from different perspectives, evidence in evidence-based medicine (EBM) can be classified according to various standards. Two common frameworks in the medical field are the “Evidence Pyramid” and the “Thomas Grading System.”

The evidence pyramid is a hierarchical representation of the strength of evidence based on study design. This framework shows the varying levels of methodological rigor associated with different research types, and is widely used to teach EBM concepts as well as to visualize the quality of medical evidence (Brighton B. et al., 2003). From bottom to top, the levels include: (1) Expert Opinion and Case Reports/Case Series; (2) Observational Studies; (3) Randomized Controlled Trials (RCTs); (4) Systematic Reviews and Meta-Analyses.

The Thomas Grading System is a recommendation framework designed to guide off-label drug use. It assesses the appropriateness of a drug for a specific clinical scenario by assigning recommendation levels (Wu and Wu, 2014). These levels are based on the strength of available evidence, such as findings from RCTs or systematic reviews, as well as the drug’s clinical relevance in practice. Unlike broader hierarchical models such as the Evidence Pyramid, the Thomas Grading System focuses more on real-world applicability, helping clinicians to make informed decisions even when the existing evidence is incomplete or inconclusive.

In China, evidence-based support for off-label drug use does not simply follow an existing classification system. For instance, when the Guangdong Pharmaceutical Association, 2024 compiled its “Off-Label Drug Use Catalog,” the evidence they relied on included: (1) drug usage methods listed on labels in foreign countries such as the United States, Europe, and Japan; (2) usage included in the *Chinese Pharmacopoeia Clinical Medication Instructions* and *Clinical Diagnosis and Treatment Guidelines*, authored by the Chinese Medical Association and published by People’s Health Publishing House; (3) endorsements by international clinical guidelines or consensus, such as those from the NCCN; (4) effectiveness ratings and a recommendation level of IIb or higher, with an evidence grade of B or above in Micromedex^®^; (5) applicability demonstrated by RCTs or meta-analyses published in top medical journals like *NEJM*, *The Lancet*, *JAMA*, *The BMJ*, or Tier 1 SCI journals in the specialty.

Another example is Article 24 of the *Administrative Measures for the Clinical Application of Antineoplastic Drugs (Trial)* released by China’s National Health Commission in 2020, which outlines a hierarchy for adopting evidence-based medical evidence when prescribing off-label drugs for cancer patients. The sequence prioritizes first the use of drugs already approved in other countries or regions, second the diagnostic and treatment standards and clinical guidelines from internationally recognized organizations, third those issued by national-level organizations, and fourth any additional evidence-based medical evidence. This structured approach ensures that off-label use adheres to rigorously validated medical standards.

At the hospital level, with the implementation of the Physician Law, an increasing number of hospitals are establishing their own off-label medication review procedures. On one hand, they apply a static classification of evidence levels based on several key factors: (1) indications listed on drug labels from the United States, Europe, Japan, or other regions; (2) the recommendation levels assigned by the Thomson Grading System in the Micromedex database; (3) recommendation levels stated in guidelines issued by medical societies or associations; (4) off-label uses documented in authoritative medical literature both domestically and internationally; and (5) the classification tiers within the evidence pyramid. By synthesizing these factors, off-label medications are categorized into high, moderate, low, and very low evidence levels (Zhang Y.T. et al., 2024).

On the other hand, different levels of evidence prompt different internal approval procedures for off-label medication use. These processes will be further discussed in the section “3.3.2 How should internal review be conducted”.

#### 4.1.3 Evidence-based support from the judicial perspective

From a judicial standpoint, in Chinese legal practice, when courts are adjudicating claims for damages resulting from off-label drug use they often delegate to appraisal institutions the task of determining whether a physician’s prescribing practices are based on sufficient evidence-based support. These institutions typically rely on authoritative domestic textbooks and clinical guidelines to make their evaluations. To date, no appraisals have been founded on foreign literature or the personal opinions or research positions of Chinese medical experts. When guidelines or textbooks support the prescribed medication methods, courts generally do not find the physician at fault for medical technical errors. Conversely, however, if there is no guidance from guidelines or textbooks on the prescribed use and dosage, appraisal institutions are likely to deem these as technical errors, leading courts to find the physician culpable based on these evaluations. Other forms of evidence-based data, although crucial in the compilation of medical textbooks and clinical guidelines, are currently ineffective in judicial settings in terms of determining whether a physician has committed a technical error in medical disputes.

This situation may arise for a couple of reasons. First, although foreign literature does not directly serve as a basis for judicial appraisals, it is considered in the compilation of Chinese textbooks and clinical guidelines, indirectly influencing Chinese medical practice. Second, the personal views and case-specific opinions of physicians often involve significant uncertainty and subjectivity, necessitating further validation and making them unsuitable as standalone standards for appraisals.

For example, in the case Chen Yunluo vs Nanjing Maternal and Child Health Hospital (Nanjing Intermediate People’s Court of Jiangsu Province, 2017), the court adopted the appraisal opinion that the hospital’s off-label use of medication, being supported by textbooks and treatment guidelines, did not constitute a technical error in medical practice. Similarly, in Xu Haie’s case against the Chinese Academy of Medical Sciences’ Fuwai Hospital (Beijing Xicheng District People’s Court, 2015) involved a medical liability dispute, with the appraisal concluding that although the medication dosage exceeded the recommended levels on the drug label, it aligned with the dosages suggested in the *Guidelines for Analgesia and Sedation Treatment in Intensive Care Unit Patients* formulated by the Critical Care Medicine Branch of the Chinese Medical Association, and thus the hospital had committed no technical error. However, in the dispute involving Li Mingxia, Li Yanqiong and others (Guangzhou Intermediate People’s Court of Guangdong Province, 2016), the appraisal institution consulted several authoritative texts including the People’s Health Publishing House’s *Internal Medicine*, *Diagnosis and Treatment Guidelines for Rheumatoid Arthritis*, *Practical Internal Medicine*, and *Newly Compiled Pharmacology*, finding no support for the physician’s off-label drug use and determining that the physician had made a medical error. Lastly, in the case Li vs Feicheng City People’s Hospital (Taian Intermediate People’s Court of Shandong Province, 2016), the physicians referred to a set of *Guidelines for Inducing Labor and Maturing the Uterus in Late Pregnancy*, which had been published in a journal. The appraisal deemed this source to have low evidential value and to lack authority, advising instead reliance on the *Clinical Technical Operation Norms–Obstetrics and Gynecology Volume*, which was organized by the Ministry of Health and authored by experts from the Chinese Medical Association. This guideline is mandatory for all medical institutions and carries official authority.

#### 4.1.4 The gap between medical and judicial practice

As previously mentioned, physicians and medical institutions apply different criteria than judicial authorities when determining what constitutes “sufficient” evidence. In medical practice, the scope of admissible evidence is considerably broader than that recognized in judicial proceedings. As a result, healthcare professionals who follow standard clinical protocols may unintentionally violate the more restrictive judicial criteria, ultimately facing liability for medical harm. To resolve this problem, it is essential to harmonize the judicial standards with the clinical standards for evidence-based off-label drug use.

### 4.2 How should informed consent be carried out?

Under the current system, informed consent in the medical field is primarily divided into two categories: consent for clinical diagnosis and treatment, and consent for procedures of a human experimental nature. Compared to consent for clinical treatments, informed consent for human experimentation entails stricter requirements. This raises the question: does off-label drug use fall under clinical treatment, or should it be seen as a form of human experimentation?

On one hand, according to many definitions off-label drug use can certainly be regarded as a part of clinical diagnosis and treatment, rather than as human experimentation. The purpose of human experimentation is not to aid the recovery of a specific patient, but rather to advance medical research for the benefit of public health (World Medical Association, 2024). Off-label drug use aims to help a particular patient recover, which does not align with the objectives of human experimentation.

On the other hand, however, off-label drug use clearly differs from standard clinical practices. Typical medical treatments are based on previously validated protocols or the use of approved medications to cure diseases. Off-label drug use lacks this established foundation, carrying an experimental aspect within the clinical setting (Beck and Azari, 1998). Therefore, it should be understood as clinical treatment with a particular experimental nature.

Given these characteristics, the informed consent requirements for off-label drug use should be more stringent than those for general clinical treatments, but less stringent than those for human experimentation. During normal clinical treatments, physicians must inform patients about several key aspects: their diagnostic results, the proposed treatment plan, potential risks, and the anticipated prognosis (Liang, 2018). However, when it comes to off-label drug use the range of information a physician should provide is much broader. In the case of Chen Yunluo vs Nanjing Maternal and Child Health Hospital (Nanjing Intermediate People’s Court of Jiangsu Province, 2017) the physician informed the patient about the treatment methods, potential risks, and possible adverse reactions, but did not disclose that the medication used fell under off-label use. The court determined that by failing to provide this information the physician did not fully meet the obligation to inform the patient, and thus bore a certain share of responsibility.

Effective informed consent for off-label drug use should encompass several essential elements (Zuo et al., 2022). It should offer a clear explanation of what off-label drug use entails, ensuring that patients understand this concept. Clinicians must inform patients about the off-label use of the proposed medication, and must explain the reasoning behind its selection. The consent process should also provide a thorough explanation of the treatment, including its potential benefits, risks, and any foreseeable side effects. Patients should be made aware of the advantages and disadvantages of the treatment, as well as any alternative therapeutic options that may be available.

By ensuring that patients are thoroughly informed about these aspects, healthcare providers will uphold ethical standards and promote patient autonomy and informed decision-making. This comprehensive approach to informed consent helps to bridge the gap between standard clinical practice and the experimental nature of off-label drug use, ultimately safeguarding patient interests while also allowing access to potentially beneficial treatments.

### 4.3 Why and how to establish an internal review procedure

#### 4.3.1 Why an internal review procedure is necessary

Article 29 paragraph two of the Physician Law imposes a procedural requirement on off-label drug use, stating that “medical institutions shall establish management systems to review the appropriateness of physicians’ prescriptions and medication orders, and strictly regulate physicians’ medication practices.” This procedural requirement exists for following reasons.

First, China’s healthcare system predominantly relies on services provided by medical institutions, with individual practitioners representing a much smaller portion of the sector (Jakovljevic et al., 2023). In medical services the medical institution enters into a medical service contract with the patient, and if a doctor’s error causes harm to the patient, the medical institution is held liable for compensation. Therefore, it is in the interest of medical institutions to establish a robust internal management process to regulate physician behavior and mitigate risks.

Second, having medical institutions review off-label drug use is essential to minimize the potential for bias on the part of individual physicians. Off-label prescribing involves some degree of experimental risk, especially when no effective or better treatment options are available. These risks can be much higher than those associated with standard clinical treatments. While giving physicians full autonomy to make off-label prescribing decisions might increase their professional independence, it also increases the likelihood of malpractice. By implementing a review process, medical institutions can help to identify and filter out higher-risk off-label treatments. This process also safeguards patients from the potential biases of individual doctors.

In practice, it is widely accepted that hospitals should have a formal review mechanism in place for off-label drug use. Failure to follow this process is generally seen as negligence in court, which can lead to liability for damages. For example, in the case of Liu Moumou vs the 988th Hospital of the Joint Logistics Support Force of the Chinese People’s Liberation Army (Zhengzhou Intermediate People’s Court of Henan Province, 2021), the court ruled that “the application for off-label drug use must be reviewed and approved by the hospital’s Pharmacy Administration Committee and Ethics Committee. Since the hospital failed to complete this procedure, its management of drug use was found to be non-compliant.” After considering other factors, the court determined that the hospital should bear 80% of the responsibility.

This case shows the importance of adhering to internal review processes for off-label drug use in medical institutions. Such mechanisms are essential for ensuring compliance with medical standards, mitigating risk, and avoiding potential legal liability. By following these procedures, hospitals not only protect themselves from legal consequences but also ensure a higher standard of care for patients.

#### 4.3.2 How should internal review be conducted

##### 4.3.2.1 Review bodies

Medical institutions review off-label drug use through two separate processes: an ethical review and a compliance review (Cai, 2024). The ethics committee is responsible for the former process, while the compliance review is conducted by the Pharmacy and Therapeutics Committee and the Medical Quality Management Committee (referred to collectively as the “two committees”), together with the Pharmacy Department and the Medical Affairs Department (referred to as the “two departments”).

The primary purpose of the ethics committee’s review is to ensure that the off-label drug use in question constitutes a treatment aimed at helping a specific patient recover, rather than being part of a clinical trial. As noted previously, off-label drug use and clinical trials share some similarities in that both employ therapies that are not yet fully validated. However, their fundamental distinction lies in their objectives. Off-label drug use, although experimental in nature, is primarily aimed at addressing the therapeutic needs of an individual patient and supporting their recovery. In contrast, clinical trials are designed to advance medical research and benefit a broader, unspecified population.

The ethics committee uses these criteria to classify the nature of the medical intervention. If it is deemed to be off-label drug use, the committee proceeds through the off-label approval process. If it is determined to be a clinical trial, however, the relevant regulations under the Good Clinical Practice (GCP) guidelines must be followed.

Once the ethics committee confirms that a particular intervention is clinical in nature rather than a trial, the “two committees” and the “two departments” will conduct a compliance review of the off-label drug use. The goal of this stage is to assess the legality, rationality, and safety of the proposed usage.

##### 4.3.2.2 Off-label drug use catalog

The comprehensive review procedures described above are not intended for every single instance of off-label drug use, since applying them universally would significantly reduce clinical efficiency. Consequently, many hospitals establish their own “Off-Label Drug Use Catalog” to streamline the process.

The Pharmacy and Therapeutics Committee in a hospital is charged with coordinating, evaluating, and ultimately finalizing this catalog. The committee consists of a multidisciplinary group of experts, including clinical pharmacists, physicians, pharmacy department heads, medical quality management personnel, and legal advisors. The catalog is developed through the following steps.

1. Evidence-Based Classification: Off-label drugs are categorized according to the quality of their supporting evidence.
2. Comprehensive Evaluation: Drugs are selected through a careful risk-benefit analysis, taking into account clinical needs, as well as the available resources and management capabilities of the institution.
3. Compliance Review: Both the legal department and the ethics committee review the proposed drugs to ensure they meet all relevant legal and ethical standards.
4. Drafting and Approval: After the working group prepares a draft catalog, it is submitted to the Pharmacy and Therapeutics Committee or the Medical Quality Management Committee for approval. Once approved, the catalog becomes official inside the hospital.

This catalog provides clear guidelines on which drugs can be used off-label, the specific conditions under which they can be prescribed, and the level of evidence supporting their use. By outlining these parameters, it helps physicians avoid the need for time-consuming searches for evidence or repeated justifications for off-label use. With this framework in place, a hospital can establish more efficient and standardized internal review procedures for the drugs listed in the catalog.

##### 4.3.2.3 Tiered review procedures for off-label drug use

For medications listed in their hospital’s Off-Label Drug Use Catalog, physicians enjoy a more efficient approval process. For example, they can obtain approval through the Pharmacy Department and the Medical Affairs Department without needing additional oversight from higher-level committees. In contrast, drugs that are not included in the catalog—typically those backed by weaker evidence or associated with greater risks—must undergo a more stringent approval procedure. After an initial review by the Pharmacy and Medical Affairs Departments, these drugs must also be evaluated by the Pharmacy and Therapeutics Committee, the Medical Quality Management Committee, and the Ethics Committee. This layered approval approach improves clinical workflow efficiency.

## 5 Conclusion

Off-label drug use remains widespread in China’s healthcare system. Despite the enactment of the Physician Law in August 2021, which established a legal framework for lawful off-label prescribing, our analysis shows ongoing non-compliance in three critical areas: evidence-based support, informed consent, and internal review procedures. The non-compliance in these areas has frequently led to legal liabilities for medical institutions. To address these challenges and enhance the lawful practice of off-label drug use, the following strategies are recommended:

(1) Integrate legal education into medical education. This will ensure that physicians are fully informed about the legal requirements for off-label prescribing.
(2) Encourage medical institutions to establish committees or working groups dedicated to evaluating the evidence for off-label drug uses, and providing guidance to physicians.
(3) Develop standardized informed consent documents specifically for off-label drug use, so that physicians can provide sufficient information for patients.
(4) Establish internal review procedure for off-label drug use.

By adopting these strategies, it should be possible to maximize the benefits of off-label drug use while also minimizing the risks. Moreover, China’s development and regulation of off-label drug use can also serve as a valuable reference for other countries facing similar challenges.

## Data Availability

The original contributions presented in the study are included in the article/supplementary material, further inquiries can be directed to the corresponding author.
